# Dynamics of the Urban Water Footprint on the Tibetan Plateau: A Case Study of Xining, China

**DOI:** 10.3390/ijerph18094566

**Published:** 2021-04-25

**Authors:** Zhirong Chen, Binghua Gong, Jiayi Jiang, Zhifeng Liu, Kelong Chen

**Affiliations:** 1 MOE Key Laboratory of Tibetan Plateau Land Surface Processes and Ecological Conservation, Qinghai Normal University, Xining 810008, China; 201947341014@stu.qhnu.edu.cn (Z.C.); ckl7813@163.com (K.C.); 2 Key Laboratory of Qinghai Province Physical Geography and Environmental Process, Qinghai Normal University, Xining 810008, China; 3 Center for Human-Environment System Sustainability (CHESS), State Key Laboratory of Earth Surface Processes and Resource Ecology (ESPRE), Beijing Normal University, Beijing 100875, China; 201711051216@mail.bnu.edu.cn (B.G.); 201811051137@mail.bnu.edu.cn (J.J.); 4 Faculty of Geographical Science, School of Natural Resources, Beijing Normal University, Beijing 100875, China

**Keywords:** urbanization, water safety, water pollution, Asian water tower, drylands, sustainable development

## Abstract

Determining the changes in the urban water footprint (*WF*) of the Tibetan Plateau is important for sustainable development within this region and in downstream regions. Taking Xining, the largest city on the Tibetan Plateau, as an example, this study quantified the changes in the *WF* of this region in the 2005–2018 period. We found that Xining’s total *WF* increased by 22.6%, from 8.9 billion to 10.9 billion m^3^ in this period. The increase in Xining’s gray WF (*WF_gray_*) resulting from the intensification of urban point-source pollution was the primary cause of the increase in its total *WF*. Xining’s *WF_gray_* from point-source pollution increased by 75.3%, from 3.1 billion to 5.4 billion m^3^. In addition, Xining’s *WF* far surpassed the amount of available water resources (*W_A_*) in this region. It is possible to prevent Xining’s *WF* from exceeding its *W_A_* only by simultaneously controlling point- and nonpoint-source pollution in the future. Thus, it is recommended that great importance be attached to the rapid increase in the *WF_gray_* of the Tibetan Plateau resulting from rapid urbanization and that effective measures be implemented to control point- and nonpoint-source pollution, so as to safeguard sustainable development within the Tibetan Plateau and in downstream regions.

## 1. Introduction

As the “Asian Water Tower” [1], the Tibetan Plateau spawns rivers that are well-known both domestically and internationally, such as the Yellow River, the Yangtze River, and the Lancang River. It is a region with concentrated lakes, glaciers, multiyear snow cover, and permafrost, and is strategically important for producing, storing, and transporting water resources in China and even Asia [2]. The Tibetan Plateau abounds with water resources, and its water-use dynamics are vitally important for its own sustainable development and that in downstream regions [3]. With rapid socioeconomic development, the Tibetan Plateau has been undergoing rapid urbanization, which has significantly affected its water consumption and water-use structure [4]. Urban water use has become an important factor that affects regional water resources [5]. Accurately assessing the state and dynamics of urban water use on the Tibetan Plateau and, on this basis, reasonably planning the allocation of water resources are crucial for facilitating sustainable development within the Tibetan Plateau and in its downstream regions [6].

In recent years, researchers in China and elsewhere have conducted a series of studies on the entire Tibetan Plateau and its urban areas to assess their state of water use. Some researchers analyzed and assessed the spatiotemporal distribution and dynamic variation in water resources within the entire Tibetan Plateau through field observations, statistical analyses, and remote sensing monitoring. However, these studies often failed to sufficiently account for the effects of urbanization and human activity on water use [7,8]. Some researchers quantitatively assessed the state of water use in urban areas of the Tibetan Plateau. However, these studies often took into consideration the dynamics of the use of water resources for direct consumption (e.g., water used for domestic and industrial purposes) but failed to take into account the needs of such areas as environments and sewage purification for water resources [9,10,11] or considered only the impact of urban water contamination on the utilization of water resources [12]. Currently, an integrated assessment of urban water use on the Tibetan Plateau remains lacking.

The water footprint (*WF*) offers an efficacious means to understand urban water use. The *WF* refers to the amount of water resources required by all the products and services consumed by a certain population (an individual, a region, a country, or the world) within a certain time [13]. Different from the conventional “water withdraw” index, the *WF* includes not only blue water but also green and gray water. The blue *WF* (*WF_blue_*) refers to the total amount of surface water and groundwater used during the manufacture of products and the provision of services. The green *WF* (*WF_green_*) refers to the amount of rainwater consumed by evapotranspiration during the manufacture of products (mainly crops) [13]. The gray *WF* (*WF_gray_*) refers to the amount of freshwater required to dilute the pollutant to meet the water-quality standards [13]. Thus, the *WF* can be used to comprehensively understand urban water use on the Tibetan Plateau, but the relevant research is still scarce.

**Figure 1:**
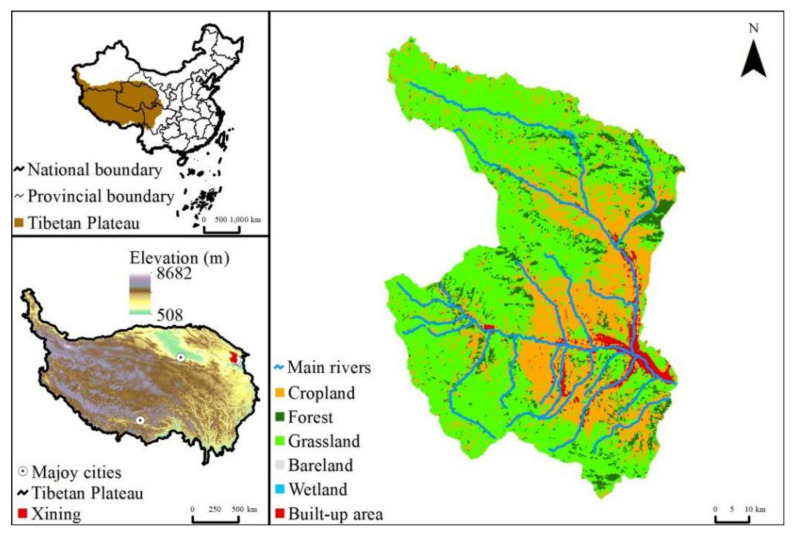
Study area.

The objective of this study was to take Xining, the largest city on Tibetan Plateau (Figure 1), as an example to understand the dynamics of *WF* under urbanization in this region. To achieve this, Xining’s total *WF*, *WF_green_*, *WF_blue_*, and *WF_gray_* were first quantified year-by-year for the 2005–2018 period in a production way. Then, the difference between the *WF* and the amount of available water resources *W_A_* was determined, and the sustainability of water use in the region was assessed. Finally, the primary causes of the changes in Xining’s *WF* and their impact on midstream and downstream regions were analyzed. The results of this study can provide a basis for thoroughly understanding the dynamics of urban water use in Xining and even the Tibetan Plateau and, thereby, offer support to facilitate sustainable water utilization in this region.

## 2. Materials and Methods

### 2.1. Study Area

Situated on the northeastern Tibetan Plateau and in the middle and lower reaches of the Huangshui River Basin, Xining (100°54′–101°56′ E, 36°13–37°23′ N) is a typical dendritic, semi-open valley city [14] that is surrounded by mountains and encompasses a total area of 7679 ha (Figure 1). With a continental plateau semiarid climate, Xining has an annual average temperature of 7.6 °C, a maximum temperature of 34.6 °C, a minimum temperature of −18.9 °C, annual average precipitation of 380 mm, and annual average evaporation of 1364 mm [15]. Xining has four districts (Chengdong, Chengxi, Chengzhong, and Chengbei), three counties (Huangzhong, Huangyuan, and Datong), a national economic and technological development zone, Chengnan New District, a biological science and technology industrial park, and Haihu New District. Xining is the only city on the Tibetan Plateau with a population exceeding one million people. By the end of 2019, Xining’s permanent resident population had reached 2.39 million people.

Xining has a well-developed, dense water system that comprises the mainstream of the Huangshui River and its 56 tributaries, with a drainage area of more than 50 ha, including the Beichuan, Nanchuan, Shatangchuan, Yaoshui, Xinachuan, and Dongxia rivers. In 2015, Xining’s water resources amounted to 1.21 billion m^3^, of which 1.19 billion m^3^ (98.35%) was surface water. In addition, in 2015, Xining consumed 567.76 million m^3^ of water [16]. In 2015, Xining discharged 99.81 million tons of urban wastewater and sewage [17]. In recent years, with the development of the Lanzhou–Xining urban agglomeration, Xining’s urbanization process has accelerated. As a result, there has been a further increase in its urban water withdrawal and wastewater discharge.

### 2.2. Data Sources

Total water withdrawal on irrigation, livestock, industrial, and domestic sectors for Xining for the 2005–2018 period, used in this study to calculate *WF_blue_*, originated from the 2005–2018 Water Resources Bulletins of Qinghai Province [16].

Meteorological, land-use/cover, and river basin division data for Xining, used in this study to calculate *WF_green_*, originated from the China Meteorological Data Service Center (http://data.cam.cn, accessed on 7 November 2020), the European Space Agency (ESA) GlobCover Dataset [18], and the Third Water Resource Survey Data produced by the Hydrological and Water Resources Bureau of Qinghai Province, respectively. Based on Yang et al.’s study [19], a threshold of 20,000 (i.e., minimum sub-river basin area ≥20,000 m^2^) was selected to divide the river basin into several sub-river basins.

Total wastewater discharge data and data for ammonia nitrogen and chemical oxygen demand (COD) discharged in wastewater for Xining, used in this study to calculate *WF* resulting from point-source pollution (*WF_gray,p_*), originated from the 2005–2018 Statistical Yearbooks of Xining and Qinghai Province [17,20]. The Class III water standard stipulated in the Standard Limits for the Basic Items of the Environmental Quality Standards for Surface Water (GB 3838-2002) was adopted (i.e., ammonia nitrogen ≤ 1.0 mg/L, COD ≤ 20 mg/L) [21]. The natural background pollutant concentration in the absence of human impact was set to 0 mg/L [22].

Digital elevation model data, used in this study to calculate Xining’s *WF* resulting from nonpoint-source pollution (*WF_gray,np_*), originated from the National Geomatics Center of China (http://ngcc.sbsm.gov.cn/, accessed on 7 November 2020).

Data for Xining’s amount of precipitation *P*, used in this study to calculate Xining’s *W_A_* and water deficit *W_D_*, originated from the 2005–2018 Water Resources Bulletins of Qinghai Province [16].

### 2.3. Methods

**Figure 2:**
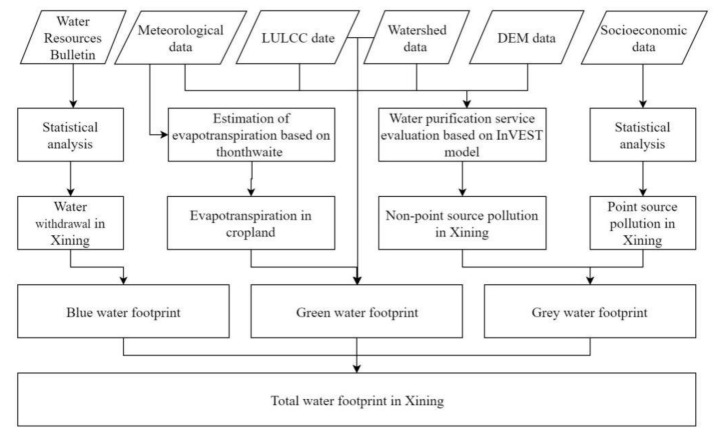
Flow chart for calculating water footprint in Xining.

Based on Liu et al.’s study [23], Xining’s *WF* is given by

(1) $$WF=WF_{blue}+WF_{green}+WF_{grey}$$

where *WF* is the total water footprint, *WF_blue_* is the blue water footprint, *WF_green_* is the green water footprint, and *WF_gray_* is the gray water footprint (Figure 2).

#### 2.3.1. Calculation of WF_blue_

The *WF_blue_* measures the amount of surface water and groundwater used for production and service purposes within a region and within a certain period of time [24]. The *WF_blue_* is calculated as follows:

(2) $$WF_{blue}=\underset{i}{\sum }WU_{i}$$

where *WU_i_* is the amount of type *i* (e.g., irrigation, livestock, and domestic) water withdrawal, which was directly obtained from the Water Resources Bulletins of Qinghai Province.

#### 2.3.2. Calculation of WF_green_

The *WF_green_* mainly refers to the total amount of rainwater consumed by evapotranspiration in cropland [25]. The *WF_green_* is calculated as follows [13]:

(3) $$WF_{green}=\sum_{i=1}^{n}ET_{cropland,i}$$

where *ET_cropland,i_* is the evapotranspiration from cropland in the *i^th^* pixel. Cropland distribution used in this study originated from the ESA GlobCover Dataset [18]. The evapotranspiration (*ET*) was estimated using the Integrated Valuation of Ecosystem Services and Tradeoffs (InVEST) model [26].

#### 2.3.3. Calculation of WF_gray_

The *WF_gray_* refers to the amount of freshwater required to dilute a certain pollutant load to meet a certain water-quality standard [13]. *WF_gray_* is calculated as follows:

(4) $$WF_{grey}=WF_{grey,p}+WF_{grey,np}$$

where *WF_gray,p_* is the water footprint resulting from point-source pollution, and *WF_gray,np_* is the water footprint resulting from nonpoint-source pollution. *WF_gray,p_* was calculated as follows [27,28]:

(5) $$WF_{grey,p}=\mathrm{Max}\left[WF_{grey,p,\left(COD\right)},WF_{grey,p,\left(NH_{3}-N\right)}\right]$$

(6) $$WF_{grey,p,i}=\frac{L_{i}}{C_{max,i}-C_{nat}}-V$$

where $WF_{grey,p,\left(COD\right)}$ is the water required to meet the standard of COD, $WF_{grey,p,\left(NH_{3}-N\right)}$ is the water required to meet the standard of ammonia nitrogen. $L_{i}$ is the emission amount of pollutant *i* (ammonia nitrogen or COD), and *C_max__,i_* is the maximum allowable concentration of ammonia nitrogen or COD in a water body, *C_nat_* is the natural background concentration of ammonia nitrogen or COD in a water body. V is the wastewater discharge volume in Xining. In this study, the values of *C_max_* for ammonia nitrogen and COD were 1 and 20 mg/L, respectively; the $\mathrm{value}\;\mathrm{of}\;C_{nat}$ was 0 mg/L [22]. 

*WF_gray,np_* was calculated as follows:

(7) $$WF_{grey,np}=\frac{L}{C_{max}-C_{nat}}$$

where L is the amount of nitrogen, which was calculated based on the InVEST model, and relevant parameters were determined based on Sharps et al.’s study [29]. *C_max_* is the maximum allowable concentration of nitrogen in water body and *C_nat_* is the natural background concentration of nitrogen in water body.

#### 2.3.4. Calculation of W_A_ and W_D_

The *W_A_* refers to the amount of water that can be used by humans without causing substantial harm to ecosystems. The *W_A_* was calculated by deducting the regional ecological water demand *W_E_* from the regional precipitation as follows [30,31]:

(8) $$W_{A}=P-W_{E}$$

where *P* is the amount of precipitation, and *W_E_* is the ecological water demand (i.e., the amount of water resources required by plants to grow in the region). *W_E_* is calculated as follows:

(9) $$W_{E}=\sum_{i=1}^{n}ET_{ecology,i}$$

where *ET_ecology,i_* is the evapotranspiration from ecological lands (e.g., forestlands, grasslands, and wetlands) in the *i^th^* pixel, calculated based on the InVEST model [26].

*W_D_*, a concept analogous to the ecological deficit, is a measure of water resource stress. The *W_D_* is the difference between the *W_A_* and the *WF*, as shown below:

(10) $$W_{D}=WF-W_{A}$$

where *W_D_* is the water deficit. When the *WF* of a region is greater than its W_A_ (i.e., *WF* > *W_A_*), there is a *W_D_*. In this case, the *W_A_* of the region is unable to meet the water demand. Conversely, when *WF* < *W_A_*, there is a water surplus. In this case, the *W_A_* of the region is able to meet the water demand.

## 3. Results

### 3.1. Xining’s WF in 2018

**Figure 3:**
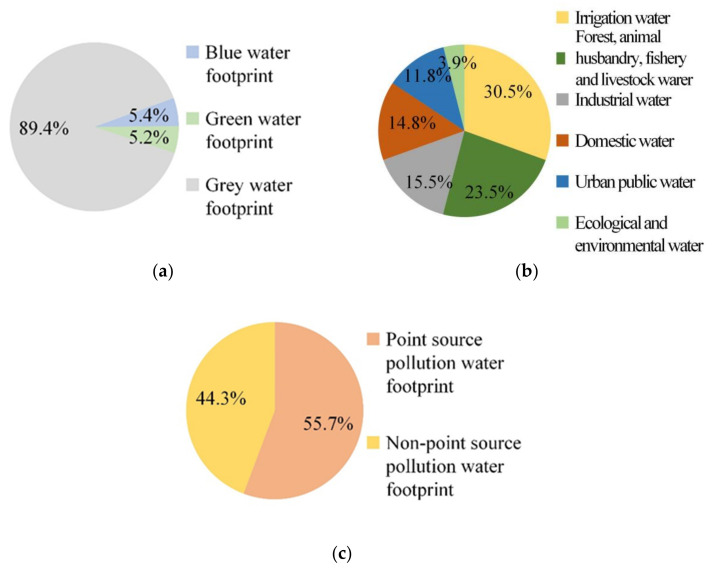
Water footprint of Xining 2018: (**a**) overall structure of water footprint; (**b**) blue water footprint structure; (**c**) gray water footprint structure.

In 2018, Xining’s *WF* exceeded the regional *W_A_*. Specifically, Xining had a *WF* of 10.9 billion m^3^ and a *W_A_* of 3.5 billion m^3^. This translates to a *W_D_* of 7.4 billion m^3^ (Table 1). Of the three types of *WF*, the *WF_gray_* was the largest contributor to the total *WF*. Xining had a *WF_gray_* of 9.7 billion m^3^, accounting for 89.4% of its total *WF* (Figure 3a). Its *WF_blue_* and *WF_green_* were 587 million and 563 million m^3^, respectively, accounting for 5.4% and 5.2% of its total *WF*, respectively. With respect to Xining’s *WF_blue_*, water used for cropland irrigation was the largest contributor (30.5%) (Figure 3b), followed by water used for forestry, fishery, and animal husbandry (23.5%). Water used for industrial, domestic, and urban public purposes accounted for 15.5%, 14.8%, and 11.8% of Xining’s *WF_blue_*, respectively. Water used for ecological and environmental purposes contributed the least (3.9%) to Xining’s *WF_blue_*. With respect to Xining’s *WF_gray_*, Xining’s *WF_gray,p_* accounted for a relatively high proportion (55.7%) of its *WF_gray_*, whereas Xining’s *WF_gray,np_* accounted for 44.3% of its *WF_gray_* (Figure 3c).

### 3.2. Dynamics of Xining’s WF in the 2005–2018 Period

**Figure 4:**
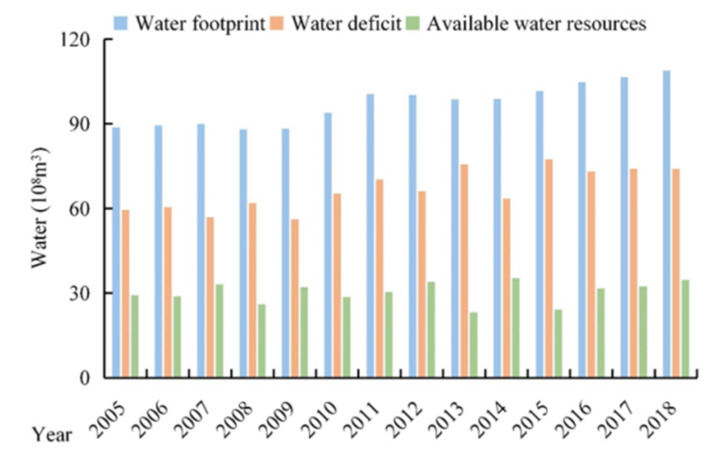
Xining’s water footprint, available water resources, and water deficit.

Overall, Xining’s *WF* displayed an increasing trend in the 2005–2018 period (Figure 4, Table 1). Xining’s total *WF* was less than 9 billion m^3^ in the 2005–2009 period but surpassed 9 billion m^3^ in the 2010–2018 period. Xining’s *WF* increased by 22.60% in the 2005–2018 period, from 8.9 billion to 10.9 billion m^3^. In the same period, Xining’s *W_A_* remained equable. Xining’s *W_D_* increased by 23.7% in the 2005–2018 period, from 6.0 billion to 7.4 billion m^3^.

**Figure 5:**
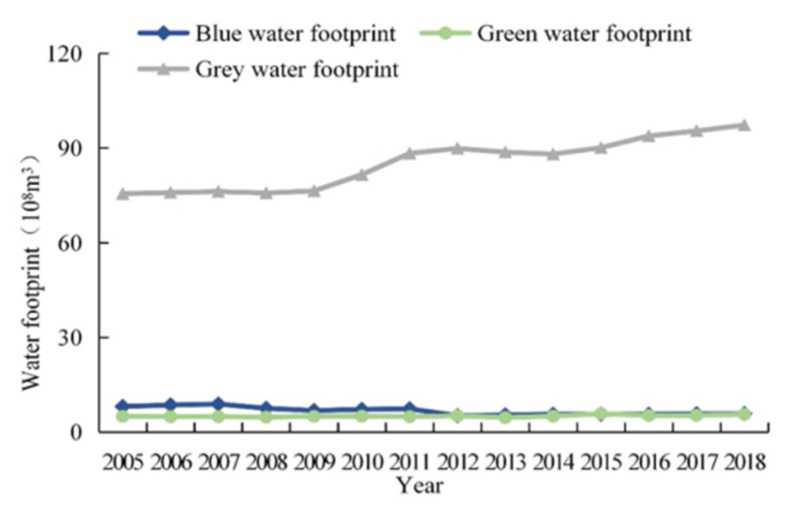
Water footprint dynamics of Xining.

Figure 5 and Table 1 show the changes in Xining’s various *WF* types in the 2005–2018 period. Overall, Xining’s *WF_gray_* increased year-on-year. Specifically, it increased by 28.8% in the 2005–2018 period, from 7.6 billion to 9.7 billion m^3^. In addition, Xining’s *WF_gray_* remained above 9 billion m^3^ each year following 2014. By contrast, there was a year-on-year decline in Xining’s *WF_blue_*. Specifically, it decreased by 27.7% in the 2005–2018 period, from 812 million to 587 million m^3^. Xining’s *WF_blue_* decreased relatively significantly after 2011. Xining’s *WF_green_* remained basically unchanged. Its minimum and maximum values (462 million and 587 million m^3^, respectively) occurred in 2013 and 2015, respectively, and differed by 26.6%.

**Figure 6:**
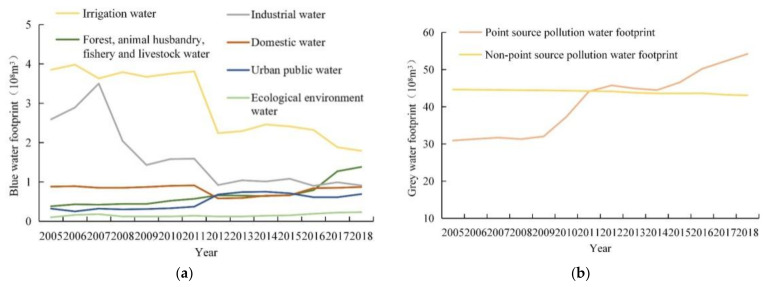
Dynamics of blue and gray water footprints in Xining: (**a**) blue water footprint; (**b**) gray water footprint.

Figure 6a and Table 2 show the changes in Xining’s various *WF_blue_* types in the 2005–2018 period. Overall, Xining’s irrigation *WF* and industrial *WF* decreased year-on-year. Specifically, they decreased by 53.5% and 64.9%, from 385 million to 179 million and 259 million to 91 million m^3^, respectively. By contrast, there were year-on-year increases in Xining’s forest, animal husbandry, fishery, and livestock *WF* and ecological and environmental *WF*. Specifically, they increased by 72.5% and 130.00%, from 38 million to 138 million and 10 million to 23 million m^3^, respectively. Xining’s domestic *WF* remained basically unchanged. Its minimum and maximum values (58 billion and 91 billion m^3^, respectively) occurred in 2012 and 2011, respectively, and differed by 26.6%. Xining’s Urban public *WF* increased from 32 million to 75 million m^3^ in the 2005–2014 period, and decreased from 75 million to 69 million m^3^ in the 2014–2018 period, which finally increased 115.6%. Figure 6b and Table 3 show the trends of Xining’s *WF_gray_*. Xining’s *WF_gray,p_* increased year-on-year. Specifically, it increased by 75.3%, from 3.1 billion to 5.4 billion m^3^. By contrast, Xining’s *WF_gray,np_* decreased year-on-year. Specifically, it decreased by 3.4%, from 4.5 billion to 4.3 billion m^3^.

## 4. Discussion

### 4.1. Comprehensive Assessment of Xining’s WF

Known as “China’s Water Tower”, the Tibetan Plateau is the source of such major rivers as the Yangtze River and the Yellow River, and it is an important ecological barrier that ensures water safety in China and even the world. Many researchers in China and elsewhere have assessed the state of water use in Qinghai Province and Xining Municipality on the Tibetan Plateau based on the *WF*. However, the relevant research considered the changes in the *WF* primarily from the perspective of domestic use and was unable to satisfactorily reflect the respective trends of various *WF* types (e.g., *WF_blue_*, *WF_green_*, and *WF_gray_*) [32,33]. Some researchers calculated the *WF_gray_* and used it to reflect the changes in water pollution in Qinghai Province. However, the relevant research focused on assessing the changes in the *WF_gray_* from the perspective of agricultural nonpoint-source pollution failed to simultaneously account for urban point-source pollution and agricultural nonpoint-source pollution and often underestimated the water deficits in this region [12,34]. For example, Sun et al. concluded that water scarcity occurred in 1.0% and 10.4% of the total areas of Qinghai Province and Tibet Autonomous Region [9], respectively. Zhang et al. noted that the Tibetan Plateau abounds with groundwater resources [35], showing an, overall, increasing trend. 

In this study, the water use in Xining in the 2005–2018 period was comprehensively assessed based on the *WF*. By calculating Xining’s *WF_blue_*, *WF_green_*, and *WF_gray_* and differentiating point- and nonpoint-source pollution when calculating its *WF_gray_*, the trend of Xining’s *WF* was sufficiently determined. It was found that Xining was facing a severe water deficit. The results of this study are consistent with those of the Third Water Resource Survey of Qinghai Province and can provide a reliable scientific basis for regional water resource management.

### 4.2. Primary Causes of the Changes in Xining’s WF

**Figure 7:**
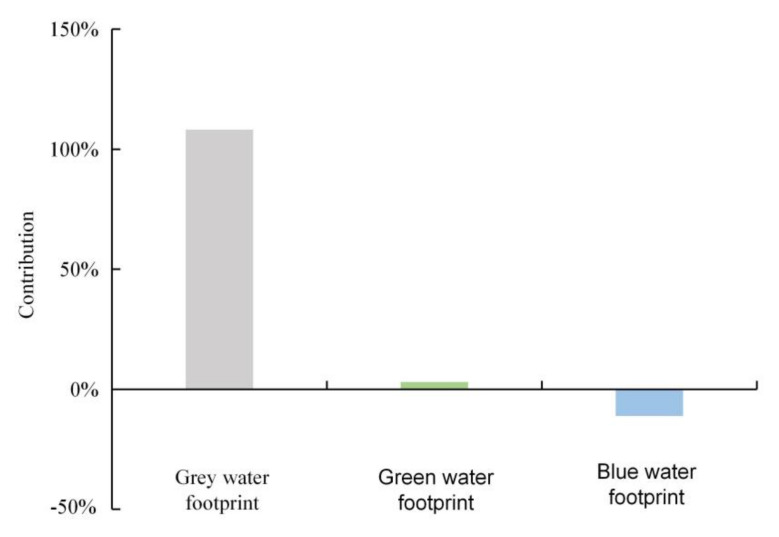
Contribution of various water footprint changes to total water footprint changes in Xining, 2005–2018.

**Figure 8:**
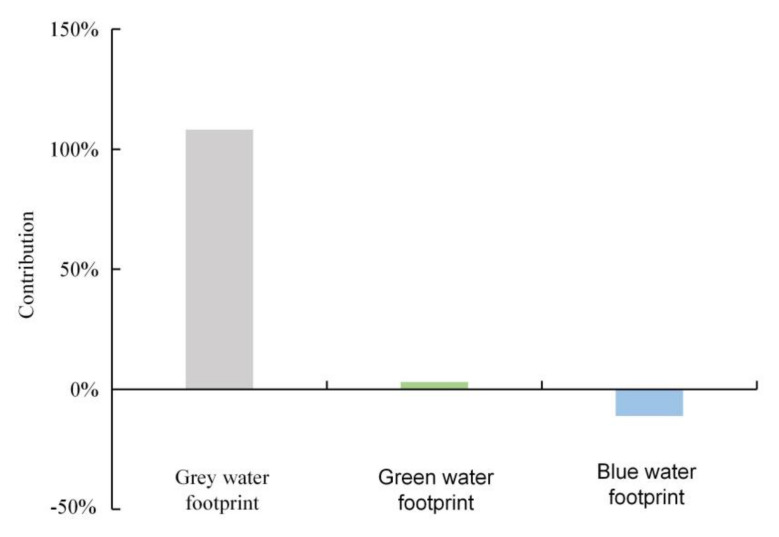
Contribution of various water uses to changes in the total water footprint of Xining, 2005–2018.

**Figure 9:**
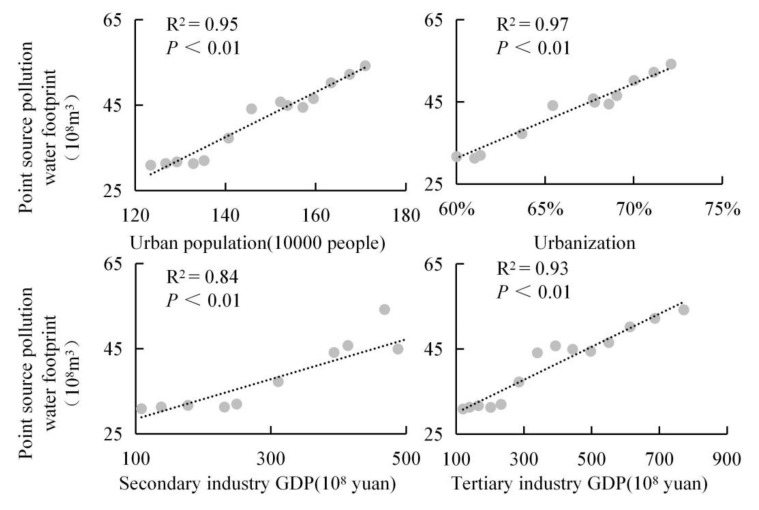
Correlation between Xining’s point source water footprint and socioeconomic development.

The increase in Xining’s *WF_gray_* was the primary cause of the increase in its total *WF* (Figure 7), and the increase in its *WF_gray,p_* was the predominant cause of the increase in its *WF_gray_* (Figure 8). Xining’s *W_Fblue_* and *WF_gray,np_* decreased year-on-year. These decreases, however, were unable to cancel out the increase in Xining’s *WF_gray,p_*. Figure 8 shows the rates of contribution of the changes in various types of water use to the changes in Xining’s total *WF*. As demonstrated in Figure 8, the rates of contribution of the changes in Xining’s *WF_gray,p_*, the amount of water used for forestry, fishery, and animal husbandry, the amount of water used for urban public purposes, and the amount of water used for public environments are positive, whereas the rates of contribution of the changes in the *WF_gray,np_*, the amount of water used for industrial purposes, and the amount of water used for cropland irrigation are negative. Figure 9 shows the correlations between Xining’s *WF_gray,p_* and socioeconomic factors. As demonstrated in Figure 9, Xining’s *WF_gray,p_* is positively correlated with its urban population (R^2^ = 0.95, *p* < 0.01), its urbanization rate (R^2^ = 0.97, *p* < 0.01), the GDP (in 100 million RMB) of its secondary industries (R^2^ = 0.84, *p* < 0.01), and the GDP (in 100 million RMB) of its tertiary industries (R^2^ = 0.93, *p* < 0.01). Thus, urbanization played an important role in the increase in Xining’s *WF_gray,p_*. 

### 4.3. Impact of the Rapid Increase in Xining’s WF on Sustainability within and outside the Region

**Figure 10:**
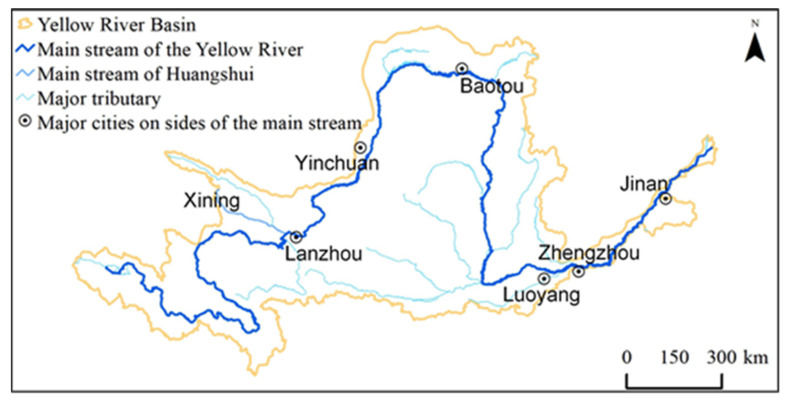
Downstream cities affected by Xining’s pollution. Note: Major cities refer to large cities with a population of one million or more.

**Figure 11:**
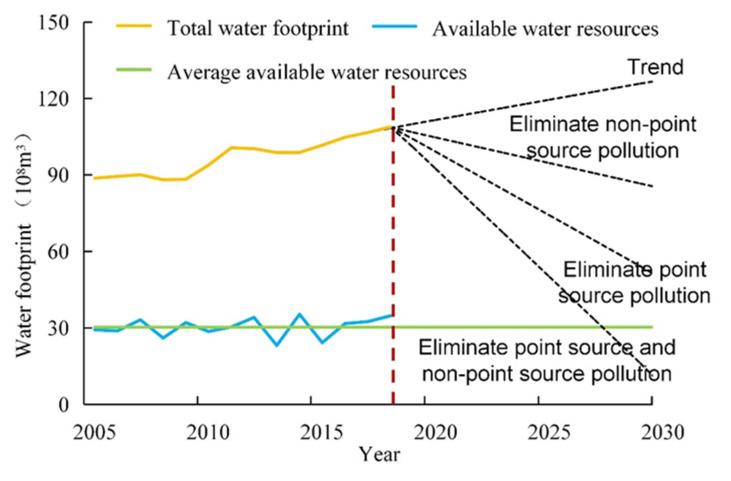
Trends in available water resources and water footprint in Xining.

The increase in Xining’s *WF_gray_* has disrupted the balance between the supply of and demand for water resources for diluting the pollutant. This has significantly affected downstream water quality and posed a threat to ecological safety in downstream rivers and water supply safety in downstream cities (Figure 10). The Huangshui River is an important water source for Xining. The region through which the Huangshui River passes is relatively densely populated and economically developed, and it is the political, economic, transportation, and cultural center of Qinghai Province. However, this region has a shortage of water resources and a low water-use efficiency [36], with an amount of water resources per person of 670 m^3^ (approximately 30% of the national average), an amount of water resources per mu of cropland of 537 m^3^ (approximately 37% of the national average), and an amount of water consumption per person of 278 m^3^ (approximately 60% of the national average). Lanzhou plays a central role in the economic zones in the upper reaches of the Yellow River and the construction of the Lanzhou–Xining urban agglomerations, and it is also an important transportation hub and a commercial and financial center in northwestern China and a major inland port city that links the West Europe Land Bridge. The Huangshui River enters Lanzhou via Haishiwan in the Honggu District and is a primary tributary of the upper Yellow River [37]. In recent years, the Huangshui River has been relatively heavily polluted, which has attracted the attention of relevant authorities [38]. Pollution in the Huangshui River significantly affects water supply safety in downstream cities, including Lanzhou. This urgently necessitates the simultaneous control of point- and nonpoint-source pollution in Xining, with a view to achieving the goal of regional sustainable development. According to the United Nations Sustainable Development Goal 6—clean water and sanitation, there is an urgent need to effectively address water scarcity and water pollution across the globe before 2030. However, if the trend seen in the 2005–2018 period continues, Xining’s *WF* will reach 12.7 billion m^3^ in 2030, and its *W_D_* will increase to 9.3 billion m^3^, 3.1 times the regional *W_A_* (Figure 11). If measures are implemented to completely eradicate nonpoint-source pollution by 2030, Xining’s *WF* will reach 8.6 billion m^3^, still 2.8 times higher than its *W_A_*. If point-source pollution is completely eliminated, Xining’s *WF* will reach 5.0 billion m^3^, and its *W_D_* will reach 2.0 billion m^3^. Xining’s *WF* will be less than its *W_A_* only when nonpoint- and point-source pollution are controlled simultaneously. To prevent Xining’s total *WF* from exceeding its *W_A_*, its WF_gray_ in 2030 needs to be reduced by 7.4 billion m^3^, 76.3% of its current *WF_gray_*.

### 4.4. Future Perspectives

In this study, the dynamics of water use in Xining were comprehensively assessed based on the *WF*. In addition, the driving forces of changes in *WF* and their effects on sustainability were also discussed. However, when calculating Xining’s *WF_gray_*, only the most key pollutants were taken into consideration. Other toxic substances (e.g., heavy metals) in trace amounts may cause more harm to the human body and require more water to dilute. Limited by data availability, the environmental flow requirements were not considered. Thus, Xining’s *WF_gray_* and water deficit may have been underestimated in this study. In addition, the model used to estimate Xining’s *WF_gray,np_* is relatively simple and requires further improvement. Nevertheless, the results of this study basically agree with those obtained by the Third Water Resource Survey of Qinghai Province. In the future, process-based models (e.g., the Soil and Water Assessment Tool) and urban metabolism models will be employed to more accurately assess the historical process and future trend of the region’s *WF*. In addition, we only calculated the water footprint and revealed the water deficit in this region, and the potential solutions as well as their feasibility and cost need further analyses.

## 5. Conclusions

Xining’s *WF* displayed an increasing trend in the 2005–2018 period. Xining’s total *WF* increased by 22.6% in the 2005–2018 period, from 8.9 billion to 10.9 billion m^3^. Its *W_A_* remained stable. Its *W_D_* increased by 23.7% in the 2005–2018 period, from 6.0 billion to 7.4 billion m^3^.

Of the changes in Xining’s various types of *WF*, the changes in its *WF_gray_* contributed significantly to the changes in its total *WF*. The increase in Xining’s *WF_gray_* resulting from intensifying urban point-source pollution was the primary cause of the increase in its total *WF*. Xining’s *WF_gray,p_* increased by 75.3% in the 2005–2018 period, from 3.1 billion to 5.4 billion m^3^. While Xining’s *WF_blue_* and *WF_gray,np_* decreased year-on-year, these decreases were unable to cancel out the increase in Xining’s *WF_gray_*. A correlation analysis between Xining’s *WF_gray,p_* and socioeconomic development showed that urban socioeconomic development was the predominant cause of the increase in Xining’s *WF_gray,p_*.

Xining’s *WF* has far exceeded the regional *W_A_*. This has caused the water quality in the middle and lower reaches of the Huangshui River, where Xining is located, to be significantly lower than the national standard and has even affected water supply safety in downstream cities, including Lanzhou. In the future, it is possible to prevent Xining’s *WF* from exceeding its *W_A_* only by simultaneously controlling point- and nonpoint-source pollution. Thus, it is recommended that great attention be paid to the rapid increase in the *WF_gray_* of the Tibetan Plateau resulting from rapid urbanization and that the government establish a *WF* management system and implement effective measures to control point- and nonpoint-source pollution, so as to safeguard sustainable development within the region and in downstream regions.

## Figures and Tables

**Table 1:** Xining’s total water footprint, available water resources, and water deficit (100 million m^3^).

Year	Blue Water Footprint	Green Water Footprint	Gray Water Footprint	Total Water Footprint	Available Water Resources	Water Deficit
2005	8.12	5.02	75.55	88.69	29.24	59.45
2006	8.60	4.90	75.90	89.40	28.86	60.54
2007	8.90	4.92	76.21	90.03	33.16	56.86
2008	7.54	4.77	75.75	88.06	26.06	62.00
2009	6.84	5.00	76.41	88.25	32.08	56.18
2010	7.20	5.05	81.58	93.84	28.58	65.25
2011	7.39	4.91	88.33	100.62	30.38	70.24
2012	5.20	5.19	89.88	100.26	34.08	66.18
2013	5.43	4.62	88.70	98.74	23.13	75.61
2014	5.65	5.06	88.07	98.78	35.33	63.45
2015	5.67	5.85	90.10	101.62	24.24	77.39
2016	5.65	5.27	93.83	104.75	31.71	73.03
2017	5.82	5.34	95.43	106.59	32.45	74.14
2018	5.87	5.63	97.27	108.77	34.75	73.55

**Table 2:** Xining’s blue water footprint.

Year	Irrigation Water	Forest, Animal Husbandry, Fishery, and Livestock Water	Industrial Water	Domestic Water	Urban Public Water	Ecological and Environmental Water
2005	3.85 (47.4%)	0.38 (4.7%)	2.59 (31.9%)	0.88 (10.8%)	0.32 (3.9%)	0.10 (1.2%)
2006	3.98 (46.3%)	0.43 (5.3%)	2.89 (35.6%)	0.89 (11.0%)	0.25 (3.1%)	0.16 (2.0%)
2007	3.63 (40.8%)	0.42 (5.2%)	3.50 (43.1%)	0.85 (10.5%)	0.32 (3.9%)	0.18 (2.2%)
2008	3.79 (50.3%)	0.44 (5.4%)	2.04 (25.1%)	0.85 (10.5%)	0.30 (3.7%)	0.12 (1.5%)
2009	3.67 (53.7%)	0.44 (5.4%)	1.43 (17.6%)	0.87 (10.7%)	0.31 (3.8%)	0.12 (1.5%)
2010	3.75 (52.1%)	0.52 (6.4%)	1.58 (19.5%)	0.90 (11.1%)	0.33 (4.1%)	0.12 (1.5%)
2011	3.81 (51.6%)	0.57 (7.0%)	1.59 (19.6%)	0.91 (11.2%)	0.37 (4.6%)	0.14 (1.7%)
2012	2.24 (43.1%)	0.66 (8.1%)	0.92 (11.3%)	0.58 (7.1%)	0.68 (8.4%)	0.12 (1.5%)
2013	2.29 (42.2%)	0.65 (8.0%)	1.04 (12.8%)	0.59 (7.3%)	0.74 (9.1%)	0.12 (1.5%)
2014	2.46 (43.5%)	0.64 (7.9%)	1.01 (12.4%)	0.65 (8.0%)	0.75 (9.2%)	0.14 (1.7%)
2015	2.41 (42.5%)	0.66 (8.1%)	1.08 (13.3%)	0.66 (8.1%)	0.71 (8.7%)	0.15 (1.8%)
2016	2.32 (41.1%)	0.79 (9.7%)	0.90 (11.1%)	0.84 (10.3%)	0.61 (7.5%)	0.19 (2.3%)
2017	1.88 (32.3%)	1.27 (15.6%)	0.99 (12.2%)	0.85 (10.5%)	0.61 (7.5%)	0.22 (2.7%)
2018	1.79 (30.5%)	1.38 (17.0%)	0.91 (11.2%)	0.87 (10.7%)	0.69 (8.5%)	0.23 (2.8%)

Note: The ratio of various types of blue water footprint to the total blue water footprint is indicated in parentheses.

**Table 3:** Xining’s gray water footprint.

Year	Point Source Pollution Water Footprint	Non-Point Source Pollution Water Footprint
2005	30.92 (40.9%)	44.63 (59.1%)
2006	31.32 (41.3%)	44.57 (58.7%)
2007	31.70 (41.6%)	44.52 (58.4%)
2008	31.30 (41.3%)	44.45 (58.7%)
2009	32.00 (41.9%)	44.41 (58.1%)
2010	37.27 (45.7%)	44.31 (54.3%)
2011	44.11 (49.9%)	44.21 (50.1%)
2012	45.74 (50.9%)	44.13 (49.1%)
2013	44.93 (50.7%)	43.77 (49.3%)
2014	44.47 (50.5%)	43.60 (49.5%)
2015	46.52 (51.6%)	43.58 (48.4%)
2016	50.21 (53.5%)	43.62 (46.5%)
2017	52.20 (54.7%)	43.22 (45.3%)
2018	54.20 (55.7%)	43.07 (44.3%)

Note: The ratio of various types of gray water footprint to the total gray water footprint is indicated in parentheses.

## Data Availability

Data is contained within the article.
